# Proteomic analysis of anti-MRSA activity of caerin 1.1/1.9 in a murine skin infection model and their *in vitro* anti-biofilm effects against *Acinetobacter baumannii*

**DOI:** 10.1128/spectrum.04520-22

**Published:** 2023-10-11

**Authors:** Pingping Zhang, Shuxian Tang, Quanlan Fu, Yuandong Luo, Junjie Li, Zhu Chen, Hejie Li, Guoying Ni, Tianfang Wang, Guoqiang Chen, Xiaosong Liu

**Affiliations:** 1 Cancer Research Institute, Foshan First People’s Hospital, Foshan, Guangdong, China; 2 Medical School of Guizhou University, Guiyang, Guizhou, China; 3 Guiyang Hospital of Stomatology, Guiyang, Guizhou, China; 4 Centre for Bioinnovation, University of the Sunshine Coast, Maroochydore BC, Queensland, Australia; University of Manitoba, Winnipeg, Manitoba, Canada

**Keywords:** caerin 1 peptide, biofilm, proteomics, skin infection

## Abstract

Caerin 1 is a family of host defense peptides with antimicrobial properties originally isolated from the Australian tree frog. Both caerin 1.1 and caerin 1.9 have been demonstrated to inhibit infections caused by multiple antibiotic-resistant bacteria, both *in vitro* and *in vivo*. In this current study, we conducted a comparison of the minimum inhibitory concentrations of caerin 1.1 and caerin 1.9 (referred to as caerin 1.1/1.9) with commonly used antibiotics against various bacteria, including *Staphylococcus aureus*, Copper-Green *Pseudomonas aeruginosa*, *Acinetobacter baumannii*, and *Streptococcus haemolyticus*. Our findings reveal that caerin 1.1/1.9 not only prevent the formation of biofilms by *A. baumannii* but also exhibit a therapeutic effect on established biofilms. Furthermore, our study demonstrates that caerin 1.1/1.9 significantly inhibit the growth of methicillin-resistant *Staphylococcus aureus* (MRSA) strains in a murine skin infection model. A quantitative proteomic analysis conducted as part of our study indicates that caerin 1.1/1.9 primarily activate oxidative phosphorylation pathways, along with several pathways associated with tissue repair and growth. These findings were observed in comparison to untreated tissues that were infected with MRSA in mice. In summary, our research outcomes suggest that caerin 1.1/1.9 exhibit the potential to serve as promising drug candidates for treating complicated antibiotic-resistant bacterial infections in humans.

**IMPORTANCE:**

Caerin 1.1 and caerin 1.9, natural antimicrobial peptides derived from tree frogs, have demonstrated the ability to inhibit the growth of antibiotic-resistant bacteria, comparable to certain widely used antibiotics. Additionally, these peptides exhibit the capacity to prevent or treat biofilms formed by bacteria in conjunction with bodily components. The mechanisms underlying their antibacterial effects were investigated through a mouse model of bacterial skin infection, utilizing proteomic analysis as a technological approach.

## INTRODUCTION

The emergence of antibiotic-resistant bacteria, which is mainly due to misuse and/or inadequate use of antibiotics, is becoming a global public health threat, not only to humans but also to animals (1). In addition, the massive scale of antibiotic use and misuse in the environment, for example, in livestock and aquaculture, results in the risk of transmission of environmental resistome across species, and eventually to humans (2). Clinically, the infections caused by resistant bacteria are more difficult to treat, normally requiring the use of more toxic and expensive drugs. In some extreme cases, bacteria show high resistance to all known antibiotics (3, 4). The antimicrobial-resistant infections grow dramatically in recent years and are directly responsible for an estimated 1.27 million deaths and associated with an estimated 4.95 million deaths worldwide in 2019, which is higher than those caused by HIV/AIDS and malaria combined (5 – 7). It is predicted that multiple drug-resistant (MDR) bacterial infections will lead to more than 10 million deaths in 2050 (8). Therefore, the development of novel and effective antibacterial agents and treatments is urgently needed.

Another major challenge in tackling bacterial infection is the formation of biofilm at the infection site (9 – 11). Biofilm refers to the complex communities of microbes that may be found attached to a surface in *Pseudomonas aeruginosa*, *Staphylococcus aureus*, and some other bacterial infection (12). Most pathogens and non-pathogens within microbial biofilms develop together as microbial communities (13), empowering them with elastic nature so that they can tolerate environmental stresses such as starvation and desiccation, to become more resilient to antibiotics (14). The treatment of mixed infections poses serious challenges in clinics as they often organize themselves as biofilm that is notoriously recalcitrant to antimicrobial therapy. Skin infection can sometimes result from multi-bacterial infection, which could lead to the formation of biofilms. Besides, nosocomial infections are confined to patients with indwelling devices used for the purpose of medical treatments, such as catheters, cardiac pacemakers, joint prosthesis, dentures, prosthetic heart valves, and contact lenses (11, 15), whose surfaces can be colonized by biofilms.

Naturally derived host defense peptides form the first line against bacteria, protozoa, fungi, and virus infection (16, 17). Antimicrobial peptides (AMPs) have the advantage of multiple functions, such as inhibition of bacteria adhesion, wide spectrum of bacterial killing, quorum sensing, and the ability to inhibit the production of extracellular polymeric substances by bacteria (18). In addition, AMPs produce less bacterial resistance than conventional antibiotics, and may show activities against MDR bacterial strains (19). More than 200 host defense peptides have been isolated and identified (20 – 23). Caerin 1.1 and 1.9 were originally isolated from the skin secretions of the Australian tree frog, the genus *Litoria*; both peptides have antimicrobial activity against a wide spectrum of Gram-positive and -negative microbial strains *in vitro* (20, 24 – 26). Caerin 1.1 and 1.9 peptides are heat resistant, and stable at low pH (5.5–7.4) and room temperature (27). The caerin peptides were predicted to interact with bacterial cell membranes via a carpet-like mechanism, whereby the peptides aggregate and orient themselves parallel to the membrane in a sheet-like arrangement followed by disruption of the bacterial cell membranes, thus killing the bacteria (28).

Caerin 1.9 has been shown to have high bioactivity against several bacteria strains with clinical significance, including MDR bacteria, such as *Staphylococcus aureus*, *Acinetobacter baumannii*, methicillin-resistant *Staphylococcus aureus* (MRSA), and *Streptococcus haemolyticus* (29). Notably, caerin 1.9 did not induce bacterial resistance after repeatedly *in vitro* culture (29). Moreover, caerin 1.1 has an additive antibacterial effect when used together with caerin 1.9. Caerin 1.1 and caerin 1.9 prepared in the form of a temperature-sensitive gel inhibit *MRSA* growth in a skin bacterial infection model of two murine strains (29). Caerin 1.1 or 1.9 immobilized on the surface of magnesium alloy materials effectively inhibit MRSA growth for over 70 h *in vitro* (30).

In the current study, we employed quantitative proteomic analysis to investigate molecular mechanisms underlying the mice skin infection with MRSA treated by caerin 1.1/1.9 temperature-sensitive gel. The significant regulation of oxidative phosphorylation, myogenesis, and adipogenesis signaling pathways were detected. In addition, we compared the minimum inhibitory concentrations (MICs) of caerin 1.1/1.9 with commonly used antibiotics against several bacteria, based on which we developed an *A. baumannii* containing biofilm model *in vitro* and showed that caerin 1.1/1.9 significantly inhibited the growth of the biofilm.

## MATERIALS AND METHODS

### Mice

Six- to 8-week-old, specific pathogen-free adult female C57BL/6 (H-2b) mice and Balb/c mice were ordered from the Animal Resource Centre of Guangdong Province and kept at the Animal Facility of the Foshan First People’s Hospital China. All mice were kept at clean condition on a 12-h light/12-h dark cycle at 22°C and the humidity was 75%. Mice were provided with sterilized standard mouse food and water. Mice were given 1% sodium pentobarbital by intraperitoneal (i.p.) injection when treatment was performed. At the end of each experiment, mice were sacrificed by CO_2_ inhalation and confirmed by ceasing breath and heartbeat.

### Peptide synthesis, caerin gel preparation, and antibiotics

Caerin 1.1 (GLLSVLGSVAKHVLPHVVPVIAEHL-NH_2_) simplified as F1, caerin 1.9 (GLFGVLGSIAKHVLPHVVPVIAEKL-NH_2_) simplified as F3, and a positive control antimicrobial peptide F-K (PLLLLLPSLLTATL-NH_2_) (31) were synthesized by Mimotopes Proprietary Limited, Wuxi, China. The purity of the peptides was >95% as determined by reverse-phase high performance liquid chromatography (HPLC) at Mimotopes. The lipopolysaccharide concentration of F1, F3, and F-K was less than 0.44 EU/mL as measured by Kinetic Turbidimetric Assay by Xiamen Bioendo Technology Co., Ltd. Sodium fusidate and dalbavancin were purchased from TargetMol, and polymyxin B was purchased from Sigma.

Poloxamer 407 (molecular weight 12,600, batch number WPAK592B) and poloxamer 188 (molecular weight 8,400, batch number WPAK539B) were purchased from Badische Anilin-und-Soda-Fabrik (Ludwigshafen, Germany). The temperature-sensitive gel containing F1/F3 was prepared as described elsewhere (32).

### Bacteria

Standard strains of *Staphylococcus aureus* (GDM1.441), MRSA (GDM1.1263), Copper-Green *Pseudomonas aeruginosa* (GDM1.443), *Acinetobacter baumannii* (GDM1.609), *Streptococcus haemolyticus* (GDM1.245), and *Escherichia coli* (GDM1.176) were purchased from the Guangdong Microbial Species Conservation Center, Guangdong, China; bacterial strain resuscitation and preservation were described elsewhere (29). All bacteria were cultured in a 37°C carbon dioxide incubator (NAPCO CO_2_ water jacket 5410-220).

### MIC

The MIC is defined as the concentration of peptide where bacterial growth is completely inhibited. The MIC of F3 against different bacteria were measured by using a micro-broth dilution method developed by the Clinical and Laboratory Standards Institute (33). Briefly, the bacterial suspension with absorbance ranging from 0.08 to 0.10 was prepared and 100 µL of the bacterial suspension to a 96 U-shaped well culture plate. The dalbavancin HCl and sodium fusidate were prepared into 328 µM, polymyxin B was prepared at 840.7 µM with phosphate-buffered saline (PBS). Concentrations of F1 and F3 and of F-K were diluted to 238 and 479.8 μM, and they were added into 96-well plates with concentration gradients of 310.5, 155.2, 77.6, 38.8, 19.4, 9.7, and 4.9 µM, respectively, with three replicates for each concentration, the equivalent volume of PBS was added as a growth control. The bacteria were cultured at 37°C for 24 h. The optical density (OD) value at 600 nm was measured with a MULTISKAN GO microplate spectrophotometer.

### The activity of caerin 1.1/1.9 against the biofilms of *Acinetobacter baumannii*

*A. baumannii* and MRSA in Muller-Hinton agar (MH) media with an OD value of 0.090 (± 0.004) were used. After 1:500 dilution with MH media, 100 µL/well of MRSA or *A. baumannii* was added into a 96-well plate, respectively. F1/F3, representing equal molar ratio of F1 and F3, was added with a final concentration ranging from 0 μM to 5.8 μM. The plate was incubated at 37°C for 48 h; the supernatants were discarded and washed with PBS three times. After fixing with 100 µL of methanol for 15 min, the methanol was discarded, and the plate was left dried naturally at room temperature. One hundred microliters of 1% Crystal Violet (LEAGENE) was then added for 30 min, followed by washing the plate with pure water three times. After the plate was completely dried, 100 µL of 33% glacial acetic acid was added and the OD value at 590 nm was measured with a MULTISKAN GO microplate spectrophotometer (Thermo Fisher Scientific, USA).

To investigate the therapeutic effects of F1/F3 against established biofilm, F1/F3 solution with different concentrations was added 24 h post the culture of MRSA or *A. baumannii*, respectively. The plate was washed with PBS and stained with Crystal Violet. The OD value at 590 nm was measured with a MULTISKAN GO microplate spectrophotometer (Thermo Fisher Scientific, USA). In another experiment, *A. baumannii* and MRSA were resuscitated cultured overnight with Bacto Brain Heart infusion, their OD values adjusted to 0.09, diluted to 104 moved into the carrier chamber slide culture. There are two prevention and treatment options. Prophylaxis adds F1/F3 with the bacterial solution. After the treatment, the bacterial membrane was formed and washed it with PBS for three times before adding it. Briefly, overnight bacterial cultures of *A. baumannii* were adjusted and inoculated into chamber slides (0030742.079, Eppendorf). After 24 h of incubation, the suspension media were discarded, and each well was washed three times with 1 mL of sterile PBS. The established biofilms were then treated with F1/F3 at the concentration of 7.5, 15, and 20 µg/mL, respectively, and incubated at 37°C for another 24 h. After washing out the non-adherent cells with PBS, the slides were then stained with two different fluorescent dyes. FM4-64 (Invitrogen) at 20 µg/mL was added and the slides were kept on ice for 1 min, or the extracellular nucleic acids were stained with 0.3 mL of propidium iodide (BD Pharmingen) at 15 µg/mL at room temperature for 15 min. After each staining step, the slides were washed with 1 mL of PBS to remove the unbound dye and dried for 15 min. All procedures were conducted in the dark. Microscopic analysis was performed using an Echo Revolve (Echo Laboratories, San Diego, CA, USA).

### Tape-stripping infection model

A tape-stripping infection model was used to investigate the antibacterial ability of F1/F3 gel (29, 34). Briefly, 6- to 8-week-old female Balb/c mice or C57BL/6 mice were anesthetized. After the removal of the fur on the back, the skin was stripped with an elastic bandage (Smith & Nephew Medical, Hull, United Kingdom). Two areas of 1 × 2 cm on both the left and right sides of the torso was tape stripped. After stripping, 10 µL of *S. aureus* (GDM1.441) suspension [5 × 10^6^ colony forming units (CFU) per milliliter] was applied for each stripped surface. Four hours after the skin infection, 20 µL of F1/F3 gel containing 4.829 mM each was applied to the left side of the stripped area, while the same amount of poloxamer gel was applied to the right side. Mice in the infection control group were given 20 µL of normal saline. Each treatment was performed twice daily for 3 consecutive days with a total of five times. On the fourth day, the stripped areas were removed and subsequently homogenized by glass homogenizer and diluted with normal saline, followed by inoculation on nutrition agar plates (LS0309, Guangzhou Dijing Microbiology Technology Co., Ltd.) for culture for 24 h before the colonies were counted.

### Protein extraction, TMT10plex labeling, and high pH reversed-phase fractionation

With the same infection model, tissue samples from the infection sites with MRSA (GDM1.1263), either treated by F1/F3 gel or untreated, as well as healthy tissue samples were collected and directly frozen in liquid nitrogen prior to protein extraction. Biological triplicates were collected for each treatment and the control. The tissue samples were homogenized thoroughly in SDS-DTT-Tris (SDT) buffer (4% [wt/vol] sodium dodecyl sulfate [SDS], 100 mM Tris-HCl pH 7.6, 0.1M DTT) at 4°C, with the total protein contents quantified using the Pierce BCA protein assay on a NanoDrop 2000 (Thermo Fisher Scientific, Bremen, Germany). The samples containing 200 µg of total protein were subjected to trypsin digestion by the filter-aided proteome preparation described elsewhere (35). Tryptic peptides were desalted on C18 Cartridges (Empore SPE Cartridges C18 (standard density), Sigma), lyophilized, and quantified on the NanoDrop 2000.

The samples containing 100 µg peptides derived from uninfected, infected, and treated tissues were labeled by TMT10plex following the manufacturer’s instruction (Thermo Scientific, Waltham, MA, USA). The labeled samples were mixed and fractionated using a Pierce high pH Reversed-Phase Peptide Fractionation Kit (Thermo Fisher Scientific, IL, USA) according to manufacturer’s instruction. All fractions were lyophilized on a SpeedVac and resuspended in 12 µL 0.1% formic acid (FA) for liquid chromatography-mass spectrometry/mass spectrometry (LC-MS/MS) analysis.

### Liquid chromatography tandem mass spectrometry analyses

A 10 µL of each sample solution was analyzed by a two-dimensional EASY-nLC1000 system coupled to a Q Exactive Hybrid Quadrupole-Orbitrap Mass Spectrometer (Thermo Scientific) using a similar method described in detail elsewhere (36, 37). Briefly, the samples were injected into the sample loading column (Thermo Scientific Acclaim PepMap100, 100 µm × 2 cm, nanoViper C18), then fractionated by the analytic column (Thermo Scientific EASY column, 10 cm, ID 75 μm, 3 µm, C18-A2). The mass spectrometer was operated in positive ion mode with MS data acquired using a data-dependent method from the survey scan (300–1,800 m/z) for higher energy collision dissociation (HCD) fragmentation. Automatic gain control target was set to 3E6, and maximum injection time to 10 ms. Dynamic exclusion duration was 40.0 s. Survey scans were acquired at a resolution of 70,000 at m/z 200 and resolution for HCD spectra was set to 17,500 at m/z 200, and isolation width was 2 m/z. Normalized collision energy was 30 eV and the underfill ratio was defined as 0.1%. The instrument was run with peptide recognition mode enabled. The mass spectrometry proteomics data have been deposited to the ProteomeXchange Consortium via the PRIDE (38) partner repository with the data set identifier PXD044425.

### Protein identification and quantification

The MS/MS data were searched against Swissprot Mouse (76,413 sequences, downloaded on 12 December 2014) database for protein identification using Mascot 2.2 (Matrix Science, London, UK) and Proteome Discoverer 1.4 software (Thermo Fisher Scientific, Waltham, MA, USA) with the following search settings: enzyme trypsin; two missed cleavage sites; precursor mass tolerance 20 ppm; fragment mass tolerance 0.1 Da; fixed modifications: Carbamidomethyl (C), TMT10plex (N-term), TMT10plex (K); and variable modifications: oxidation (M), TMT10plex (Y). The results of the search were further submitted to generate the final report using a cut-off of 1% false discovery rate (FDR) on peptide levels and only unique peptides were used for protein quantitation. All peptide ratios were normalized by the median protein ratio, and the median protein ratio was one after the normalization. The protein showing a fold change (FC) ≥1.2 (up-regulation ≥1.2 or down-regulation ≤0.83) compared to the untreated group and the *P*-value <0.05 were considered significantly regulated by the treatment and included in further analysis.

### Protein-protein interaction (PPI) analysis

Interactions among significantly regulated proteins were predicted using STRING (39). All resources were selected to generate the network and “confidence” was used as the meaning of network edges, and the required interaction score of 0.400 was selected for all PPI, to highlight the confident interactions. Neither the first nor second shell of the PPI was included in this study. Protein without any interaction with other proteins was excluded from displaying in the network.

### Gene ontology, Kyoto Encyclopedia of Genes and Genomes (KEGG) pathway, and gene set enrichment analysis (GSEA) analysis

The enrichment of biological processes and KEGG pathways (40) by the treatments with respective to untreated and control group were analyzed. The genes corresponding to the proteins differentially expressed in three groups were analyzed by GSEA with *P*-value <0.05 using GSEA v4.1.0 (41, 42).

### Statistical analysis

Paired student *t*-test statistical analysis was performed to evaluate the *in vivo* bacteria growth inhibition using GraphPad Prism 7 software. All experimental data were analyzed, and graphs were plotted in the same software. The significant means were determined at the probability level of 0.05.

## RESULTS

### Comparison of MICs against various bacteria for F1, F3, and different antibiotics

**Fig 1:**
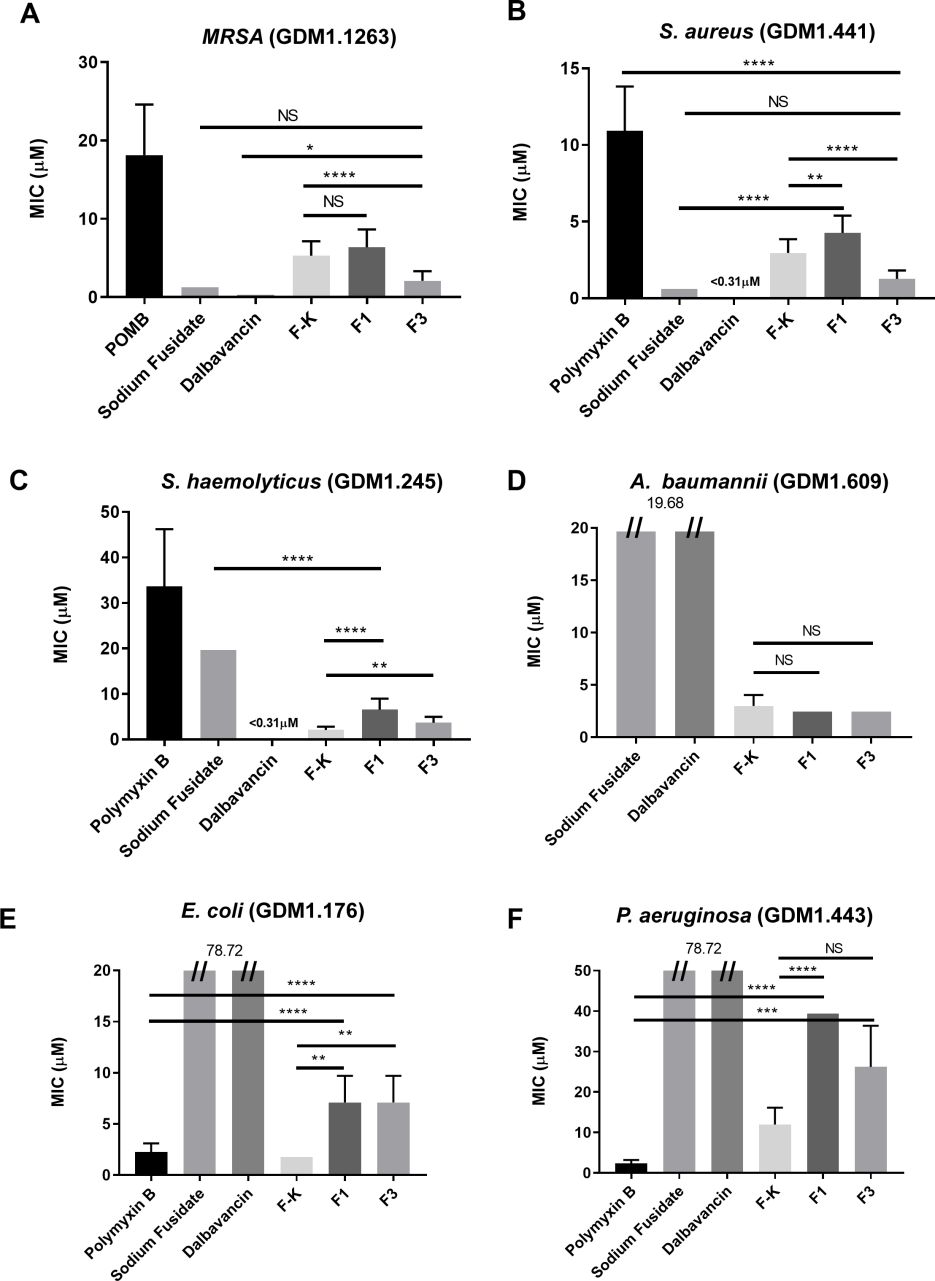
The comparison of MICs against different bacteria between F1, F3, and several antibiotics. The MICs of F1 and F3, polymyxin B, sodium fusidate, dalbavancin, and F-K peptide, against *S. aureus* (GDM1.441), *P. aeruginosa* (GDM1.443), MRSA (GDM1.1263), *A. baumannii* (GDM1.609), *S. haemolyticus* (GDM1.245), and *E. coli*. The MICs against MRSA (**A**), *S. aureus* (**B**), *S. haemolyticus* (**C**), and *A. baumannii* (**D**), *E. coli* (**E**), and *P. aeruginosa* (**F**).

Previously, we showed that F1 + F3 was able to inhibit multiple antibiotic-resistant bacteria infection in a murine skin infection model (29). We now compared the MICs of caerin 1.9 against *S. aureus*, *P. aeruginosa*, MRSA, *A. baumannii*, *S. haemolyticus*, and *E. coli*, with respect to four other antibiotics that are commonly used in clinics (Fig. 1). It was evident that caerin peptides exhibited high inhibitory activity against the growth of Gram-positive bacteria, including MRSA, *S. aureus*, *S. haemolyticus*, and *A. baumannii*. The MICs of F1 or F3 against MRSA, *S. aureus* were superior to polymyxin B but inferior to sodium fusidate or Ddalbavancin (Fig. 1A). The MICs of F3 on MRSA and *S. aureus* were significantly lower than that of another antimicrobial peptide K-F (Fig. 1A and B). The MICs of F1 and F3 inhibiting *S. haemolyticus* were better than polymyxin B and sodium fusidate, yet higher than dalbavancin (Fig. 1C). Dalbavancin and sodium fusidate had no inhibitory activity on *A. baumannii,* while F1, F3, and F-K showed comparable inhibition (Fig. 1D). The inhibitory effect of F1 and F3 against Gram-negative bacteria was comparatively low yet still present. For example, both F1 and F3 exhibited suppression of the growth of *E. coli* and *P. aeruginosa* at high concentrations, while dalbavancin and sodium fusidate were incapable of stopping these bacterial growth. F-K peptide, however, was the best at inhibiting these two strains of bacteria (Fig. 1E and F).

### F1/F3 inhibited the formation of MRSA and *A. baumannii* biofilm

**Fig 2:**
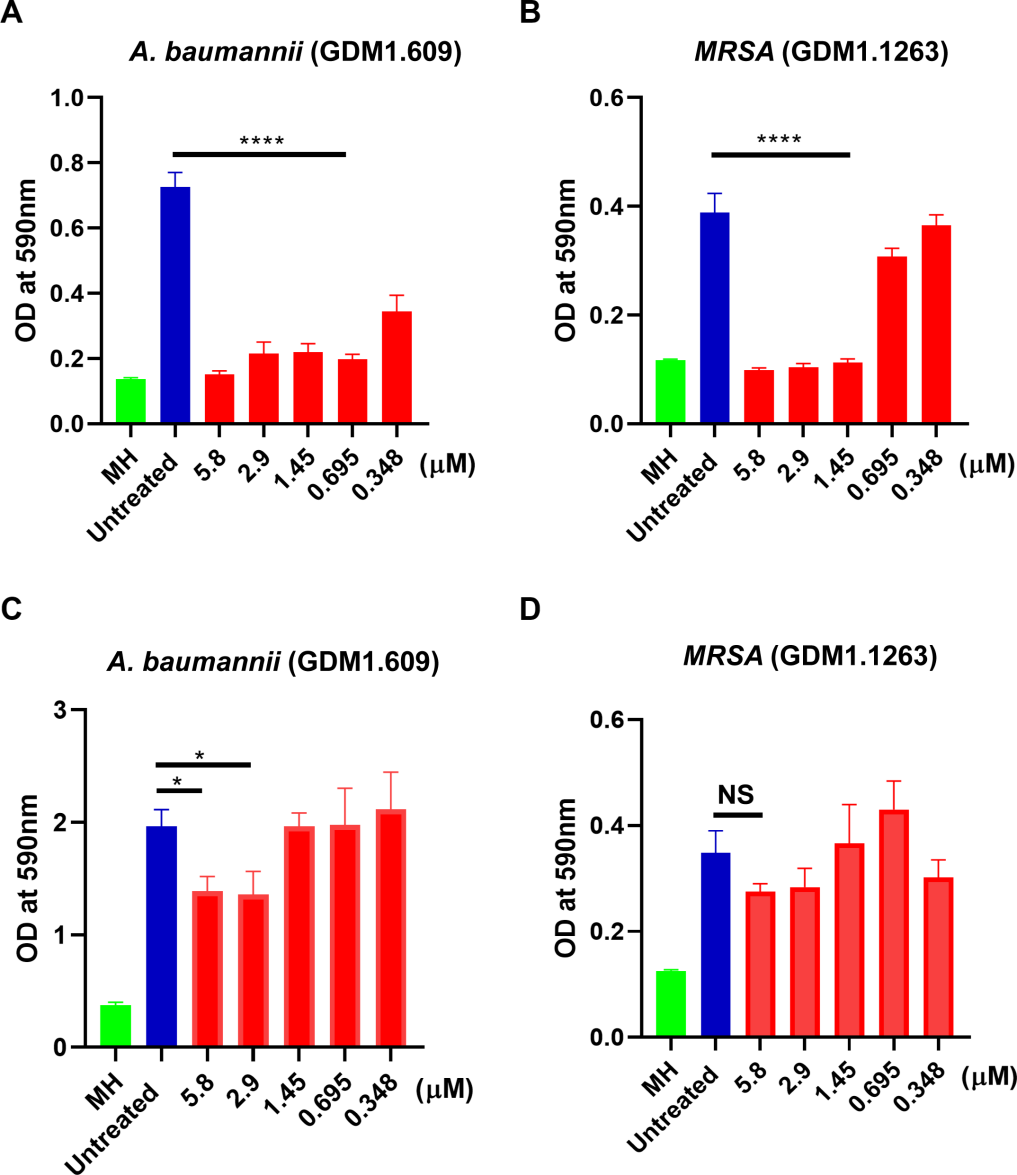
F1/F3 demonstrated anti-biofilm effects against both *A. baumannii* (GDM1.609) and MRSA (GDM1.1263). These effects included the inhibition of biofilm formation for both *A. baumannii* (**A**) and MRSA (**B**). Furthermore, F1/F3 exhibited therapeutic efficacy against established biofilms of *A. baumannii* (**C**) and MRSA (**D**).

Next, we investigated whether F1/F3 are able to influence biofilm formation and whether they have therapeutic effects on established biofilm. As shown in Fig. 2, F1/F3 have the capability to inhibit the biofilm formation of MRSA and *A. baumannii*. F1/F3 at the concentration of 347.672 nM inhibited the formation of biofilm by *A. baumannii* (Fig. 2A). To inhibit the biofilm formation by MRSA, the concentration of F1/F3 was above 1.45 µM (Fig. 2B). We also assessed whether F1/F3 can treat established biofilm. Once the biofilm containing *A. baumannii* was formed at 24 h post-culture, F1/F3 inhibited further development of the biofilm at a concentration higher than 1.45 µM, which was almost fourfold higher than that prevented the formation of biofilm (Fig. 2C). However, F1/F3 had no effect on established biofilm by MRSA (Fig. 2D).

**Fig 3:**
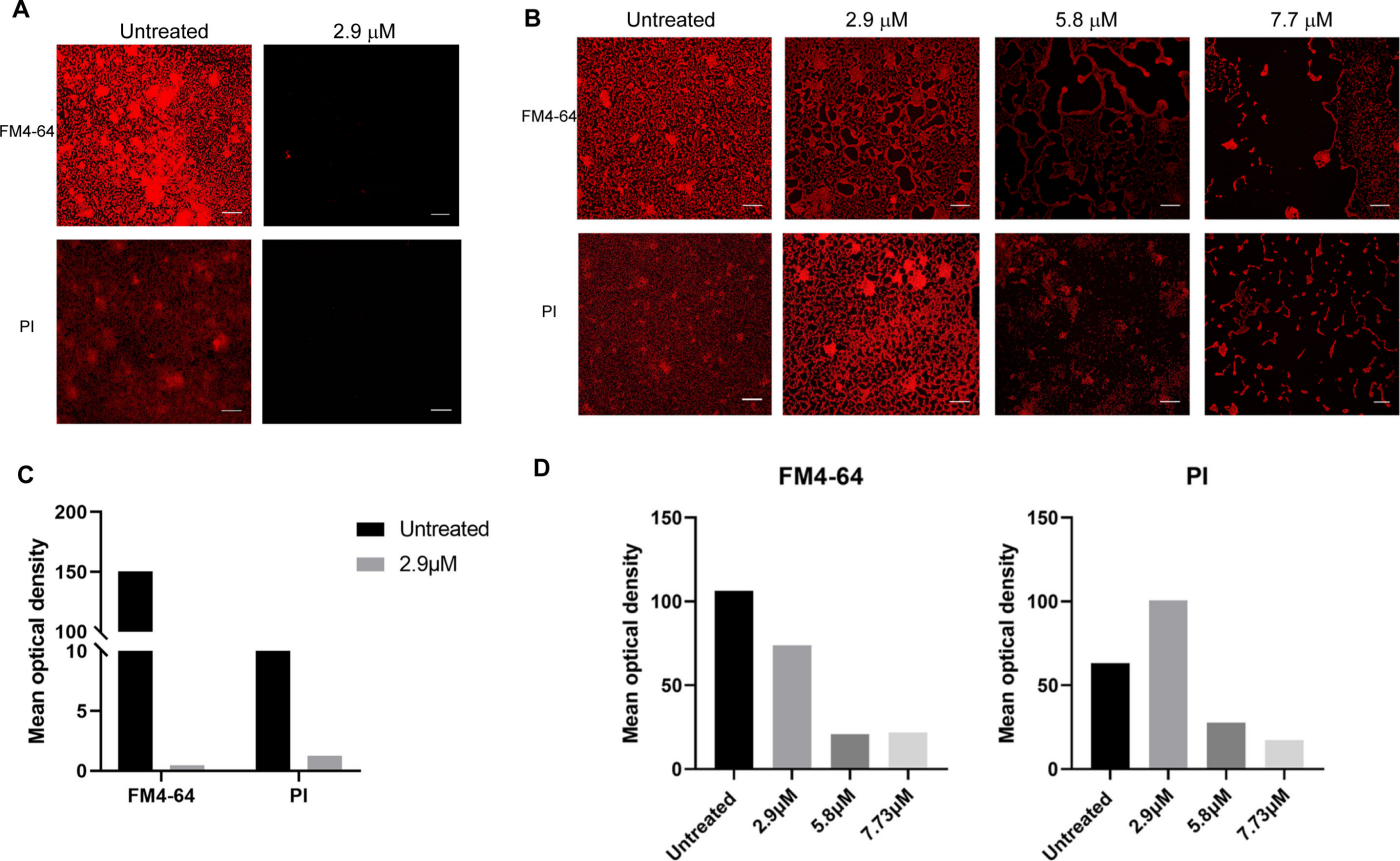
Microscopic evaluation of the impact of F1/F3 on established biofilms of *A. baumannii* (GDM1.609). (**A**) F1/F3 prevented the formation of *A. baumannii* biofilm. The biofilm of *A. baumannii* formed on a chamber slide surface was treated with F1/F3; after 24 hours of treatment, the biofilm was stained with FM4-64 to visualise the plasma membrane and with PI to visualise nucleic acids using a ECHO Revolve Microscope. (**B**) F1/F3 significantly reduced formed biofilms of *A. baumannii*. The biofilms were formed on a chamber slide surface; after 24 hours, these biofilms were treated with different concentrations of F1/F3. The displayed images were captured at a 20× magnification. (C) Mean optical density of FM4-64 and PI labels in untreated and F1/F3 treated biofilms during its formation shown in (A). (D) Mean optical density of FM4-64 and PI labels in untreated biofilm and formed biofilm samples treated with F1/F3 at 2.9, 5.8, 7.7 µM as displayed in (B).The bar length in the images corresponds to 3.45 µm.

To confirm the above results, *A. baumannii* was added to the chamber slide together with F1/F3 at different concentrations. At 24 h post-culture, FM4-64 and propidium iodide (PI) were used to stain the cell membrane and the extracellular nucleic acid, respectively. The results showed that F1/F3 prevented the formation of *A. baumannii* biofilm at 2.897 µM (Fig. 3A). For the treatment of biofilm, *A. baumannii* was inoculated into the chamber slide and cultured overnight. After the biofilm was formed, F1/F3 were added and incubated for 24 h, and then the above dyes were used. It was clear that the anti-biofilm activity of F1/F3 against *A. baumannii* was positively correlated with the concentration (Fig. 3B), which was consistent with the above results showed in Fig. 2. The reduction of mean optical density during the biofilm formation inhibition was nearly 100% and 90% for plasma membrane and nucleic acids, respectively (Fig. 3C). In terms of treating formed biofilm, the mean optical density of FM4-64 decreased progressively with increasing concentration of F1/F3, while the abundance of nucleic acids increased at 2.9 µM of F1/F3, followed by a significant decrease in a dose-dependent manner (Fig. 3D).

### F1/F3 inhibited *S. aureus* growth in a murine skin infection model

**Fig 4:**
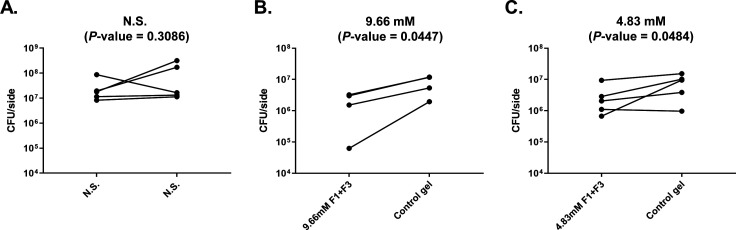
The effectiveness of F1/F3 gel in inhibiting the growth of *S. aureus* (GIM1.1441) on tape-stripped infection in Balb/c mice was assessed. This involved comparing the bacterial counts between treatments using two different concentrations of the F1/F3 gel (9.658 mM and 4.829 mM) and a blank gel, with the infected areas treated with a similar amount of normal saline serving as the control group. Initially, approximately 5 × 10^4^ CFU of bacteria were inoculated on each wound, which were then treated with either caerin gel or control gel for 3 days. Each line in the graph represents the paired comparison of the number of bacteria (CFU) between the right- and left-stripped areas per mouse. The three groups depicted are (**A**) normal saline treatment group (*n* = 5), (**B**) 9.658 mM caerin gel treatment group (*n* = 4), and (**C**) 4.829 mM F1/F3 gel treatment group (*n* = 5). These results presented are from one representative experiment out of two independent experiments conducted. The statistical significance was assessed using a paired *t*-test, where a *P*-value <0.05 was considered to indicate a significant difference between groups.

We further investigated the *in vivo* antibacterial activity of F1/F3 gel against *S. aureus* in the same tape-stripped model. The suppression of *S. aureus* growth was displayed after the gel treatment with respect to the group treated with control gel (Fig. 4). Normal saline provided no inhibitory effect for the growth inhibition of *S. aureus* (Fig. 4A), while inhibition was observed either at 9.658 mM (*P*-value = 0.0447) (Fig. 4B) or at 4.829 mM (*P*-value = 0.0484) (Fig. 4C) for caerin gel. The result indicated that F1/F3 in the form of temperature-sensitive gel can inhibit *S. aureus* growth in the skin infection model.

### Proteomic analysis reveals the activation of oxidative phosphorylation in the murine skin infection by MRSA with the treatment containing F1/F3

**Fig 5:**
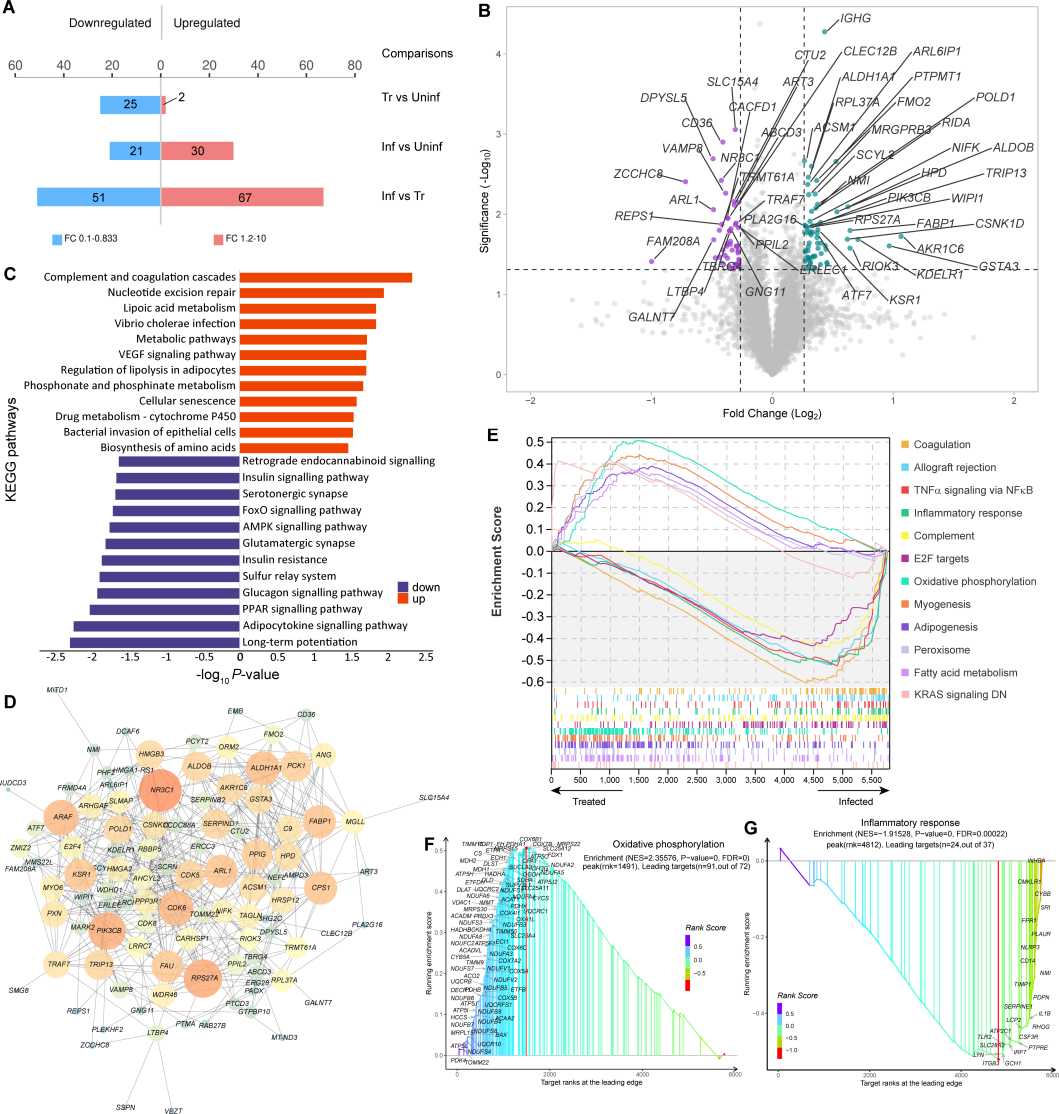
Comparison of the protein profiles of uninfected (Uninf), infected (Inf), and treated (Tr) mice using TMT10plex quantitative proteomics. (**A**) The number of DEPs with FC >1.2 and *P*-value <0.5 identified in the comparisons between Uninf, Inf, and Tr groups. (**B**) The volcano graph shows the DEPs of the Inf group with respect to the Tr group. (**C**) The enrichment analysis of KEGG pathways represented by the DEPs up- or down-regulated in the Inf group, respectively. (**D**) Protein-protein interaction network among the DEPs identified when comparing the Inf and Tr groups. (**E**) GSEA illustrates the top 12 hallmark pathways that are enriched in the Inf and Tr groups, respectively. (**F**) The ranking of the DEPs is presented based on their relevance to the enrichment of specific pathways, such as “oxidative phosphorylation” and “inflammatory response.”

Previously, we demonstrated that F1/F3 in the form of temperature-sensitive gel were able to inhibit MRSA growth in a murine skin infection model (29). To investigate the potential molecular mechanism underlying this treatment, we performed TMT10plex-labeled proteomic analysis to compare the proteome profiles of uninfected (Uninf), infected (Inf), and treated (Tr) skin samples (Fig. S1). A total of 5,859 proteins were identified across all samples, supported by 32,887 unique peptides. There were high number of differentially expressed proteins (DEPs, unpaired *t*-test *P*-value <0.05) between the Tr and Inf groups, with 67 up-regulated and 51 down-regulated (Fig. 5A; Table S1). Several DEPs known to play important roles in active immune response were up-regulated in the Tr group, including *Cd36*, *Fm208a*, *Clec12b*, and *Ptma*, while several others were up-regulated in the Inf group, such as *Riok3*, *Hmgb3*, *Zdhhc6*, *Orm2*, and *C9* (Fig. 5B). Multiple DEPs associated with various metabolisms were up-regulated in the Inf group, for instance, *Fmo2*, *Rida*, *Ampd3*, and *Ascm1*. The difference between the protein profiles of Inf and Uninf group was comparatively smaller, with fewer DEPs present. There were only two proteins, *Csnk1d* and *Gulo*, significantly up-regulated in the Tr with respect to the Uninf group. The KEGG pathway analysis based on the DEPs between the Inf and Tr groups showed that several metabolism pathways were enriched in the Inf group, such as the metabolism of lipoic acid, phosphonate, and phosphinate, “drug metabolism-cytochrome P450,” and more general “metabolic pathways” (Fig. 5C). Notably, two pathways relevant to bacterial infection were also enriched, including “vibrio cholerae infection” and “bacterial invasion of epithelial cells.” The pathways over-represented by the DEPs down-regulated in the Inf group (i.e., up-regulated by the treatment) included one pathway directly related to immune response, “adipocytokine signaling pathway.” Interestingly, several other enriched pathways were relevant to nervous system.

There were significant interactions among the DEPs between the Inf and Tr groups (Fig. 5D; Fig. S2). Most DEPs were predicted to interact with another node protein directly or within two steps, thus showing increasing closeness centrality along with the number of neighbor DEPs increased. Several proteins associated with transcription, cell growth, and development were among the nodes with high degrees, such as *NR3C1*, *RPS27A*, *ALDH1A1*, *PIK3CB*, *CDK6*, and *FAU*. The DEPs playing roles in immune response had relatively less interactions. Several immune response relevant hallmark pathways were significantly activated in the Inf compared with the Tr group, including “IFNγ response” (*P*-value = 0.01854), “allograft rejection” (*P*-value = 0), “TNFα signaling via NFκB” (*P*-value = 0), “inflammatory response” (*P*-value = 0), “complement” (*P*-value = 0), and “IL6/JAK/STAT3 signaling” (*P*-value = 0.02353) (Fig. 5E; Table S2). In contrast, the pathways associated with cell and tissue development were highly activated in the Tr group, such as “myogenesis,” “peroxisome,” and “adipogenesis.” Notably, “oxidative phosphorylation” was the most activated pathway supported by several up-regulated DEPs in the Tr group (Fig. 5F). The activation of “inflammatory response” pathway was mainly attributed to the up-regulation of *INHBA*, *CMKLR1*, *CYBB*, *SRI*, *FPR1*, *PLAUR*, *NLRP3*, and *CD14* in the Inf group (Fig. 5G). These observations indicated that a longer-term activation required for tissue repair and the return to homeostasis was present in the Tr group, whereas it was a pro-inflammatory state dominant in the tissue of the Inf group.

## DISCUSSION

In this study, we showed that the MICs of F1/F3 against a variety of bacteria were compatible with commonly used antibiotics, and discovered that the temperature-sensitive gel containing F1/F3 effectively cured skin infection by *S. aureus* in a murine model. We also demonstrated that F1/F3 were able to prevent the formation of biofilm by MRSA and *A. baumannii*, respectively; in addition, they had therapeutic effects against established biofilm containing *A. baumannii* in a murine infection MRSA infection model. The quantitative proteomic analysis suggested that pro-inflammatory immune response was dominant in the infected tissue, while the oxidative phosphorylation signaling and several pathways promoting tissue repair and growth were highly activated by the treatment with F1/F3 gel.

Sodium fusidate and dalbavancin showed better performance than F1 or F3 at inhibiting MRSA and *S. aureus*, while F1 or F3 are better than F-K peptide, an antimicrobial peptide under stage III clinical trial, at inhibiting the growth of these bacteria strains. However, the prevalence of sodium fusidate-resistant MRSA or *S. aureus* is increasing (43), and dalbavancin-resistant MRSA begin to merge (44). Therefore, new antimicrobial antibiotics as caerin peptides may provide alternative ways to combat these antibiotic strains of MRSA or *S. aureus*. Previously, we demonstrated that F1/F3 prepared in the form of a temperature-sensitive gel inhibit MRSA growth in a skin bacterial infection model of two murine strains (45), while we observed similar phenomenon that they also inhibit *S. aureus* growth in the same model in this study.

The biofilm poses severe problem due to its resistance to antibiotic treatment (46). *A. baumannii* is an important nosocomial pathogen that is responsible for a wide range of human infections. Most of the *A. baumannii* isolated from patients can form biofilm (47 – 49). The ability of biofilm formation leads to *Acinetobacter*’s easy survival and transfer in hospital environment, which includes various biotic and abiotic surfaces, e.g., vascular catheters, cerebrospinal fluid shunts, or Foleys catheter (50, 51). *A. baumannii* was sensitive to F1 and F3; the MICs were both at around 5 µM (Fig. 2). Moreover, F1/F3 were able to prevent the formation of biofilm by MRSA and *A. baumannii* at the concentration of 1.9 and 1.45 µM, respectively (Fig. 4A and B). F1/F3 also had therapeutic effect against established biofilm of *A. baumannii*, at the concentration of 2.897 µM, while they lost their ability to influence the established biofilm of MRSA (Fig. 4C and D). We have recently immobilized F1 and F3 on the surface of magnesium alloy, which showed the capability to inhibit MRSA growth (52). Thus, F1 and/or F3 may be applied onto the surface of medical device to prevent the formation of biofilm caused by *A. baumannii*. We are currently investigating the mechanism against *A. baumannii*-associated biofilm.

The proteomic analysis found that the “complement and coagulation cascades” pathway was enriched by the DEPs up-regulated in the infected group, which is accorded with the GSEA results. It has been recognized that the activation of coagulation pathway is beneficial for bacterial infections (53, 54), which indicated that the over-representation of this pathway was likely related to the bacterial infection introduced to the murine model. This was also supported by the enrichment of “vibrio cholerae infection” and “bacterial invasion of epithelial cells.” The bacterial infection induced the reaction of the innate immune system, which was reflected by the enrichment of several immune response-relevant pathways by the GSEA. Oxidative phosphorylation serves as a crucial metabolic pathway in charge of energy production in many cells, including immune cells. For example, oxidative phosphorylation is vital for natural killer (NK) cell receptor-activated cell cytotoxicity and effector functions (55). It has previously been found that the inhibition of oxidative phosphorylation in M1 macrophages generates bactericidal reactive oxygen species (ROS), while it is largely required by M2 macrophages for tissue repair and the return to homeostasis (56). Considering there are only two DEPs up-regulated in the Tr relative to the Uninf groups, as well as no immune response-relevant signaling was activated in the Tr compared with the Inf group, we thus speculate that oxidative phosphorylation activated largely in the Tr group supported the tissue growth and wound healing. This indicates that the antibacterial process had largely concluded by the time the tissue samples were collected. Previous studies have consistently demonstrated that host defense peptides exhibit limited toxicity toward normal mammalian cells (23, 57, 58). This suggests that the effects of these peptides on uninfected mice are likely to be minimal. Nevertheless, a detailed investigation of the effects of caerin peptides on healthy skin tissue is warranted if considering the gel for clinical application. There is limitation in proteomic analysis that the adjusted *P*-values, with an FDR threshold of 0.05 using the Benjamini-Hochberg procedure, are greater than 0.05 (Table S1). This suggests that, in the context of a single hypothesis test, the observed DEPs between conditions (i.e., Tr, Inf, and Uninf) are statistically significant at the 0.05 significance level. However, we acknowledge that the significance of the finding is not robust to multiple testing correction and may require validation in subsequent experiments with more replicates.

Taken together, F1/F3 have similar *in vitro* efficacy against MRSA and *S. aureus* compared with dalbavancin and sodium fusidate and are effective against the biofilm formed by *A. baumannii*. F1/F3 inhibits the growth of MRSA and *S. aureus* in a murine skin infection model, and activate oxidative phosphorylation and pathways supporting tissue repair and wound healing in damaged tissue, in addition to killing the bacteria. Thus, F1/F3 may provide an alternative way to combat skin bacterial infection in human, which warrants further investigation.

## Data Availability

The mass spectrometry proteomics data have been deposited to the ProteomeXchange Consortium via the PRIDE(38) partner repository with the dataset identifier PXD044425.

## SUPPLEMENTAL MATERIAL

Fig. S1 spectrum.04520-22-s0001.tif Hierarchical clustering of differentially expressed proteins identified between (A) Inf vs Tr, (B) Inf vs Uninf, and (C) Uninf vs Tr.

Fig. S2 spectrum.04520-22-s0002.tif Enrichment analysis of gene ontology and protein-protein interaction analysis.

Fig. S3 spectrum.04520-22-s0003.tif Statistical analysis of the PPI network shown in Fig. 1D.

Fig. S4 spectrum.04520-22-s0004.tif Statistical analysis of the PPI network shown in Fig. S2D.

Supplemental legends spectrum.04520-22-s0005.docx Legends for Fig S1 to S4 and Tables S1 to S3.

Table S1 spectrum.04520-22-s0006.xlsx Comparison of protein profiles of different groups and supporting peptides identified by LC-MS/MS.

Table S2 spectrum.04520-22-s0007.xlsx Supporting peptides for proteomic results.

Table S3 spectrum.04520-22-s0008.xlsx GSEA of the hallmark pathways enriched in the treatment with respect to the infected group.

ASM does not own the copyrights to Supplemental Material that may be linked to, or accessed through, an article. The authors have granted ASM a non-exclusive, world-wide license to publish the Supplemental Material files. Please contact the corresponding author directly for reuse.
