# Supplementary figures and images for: Proteomic analysis of anti-MRSA activity of caerin 1.1/1.9 in a murine skin infection model and their in vitro anti-biofilm effects against Acinetobacter baumannii
Source: Microbiol Spectr. 2023 Oct 11;11(6):e04520-22. doi: 10.1128/spectrum.04520-22 (PMC10714828; doi:10.1128/spectrum.04520-22)

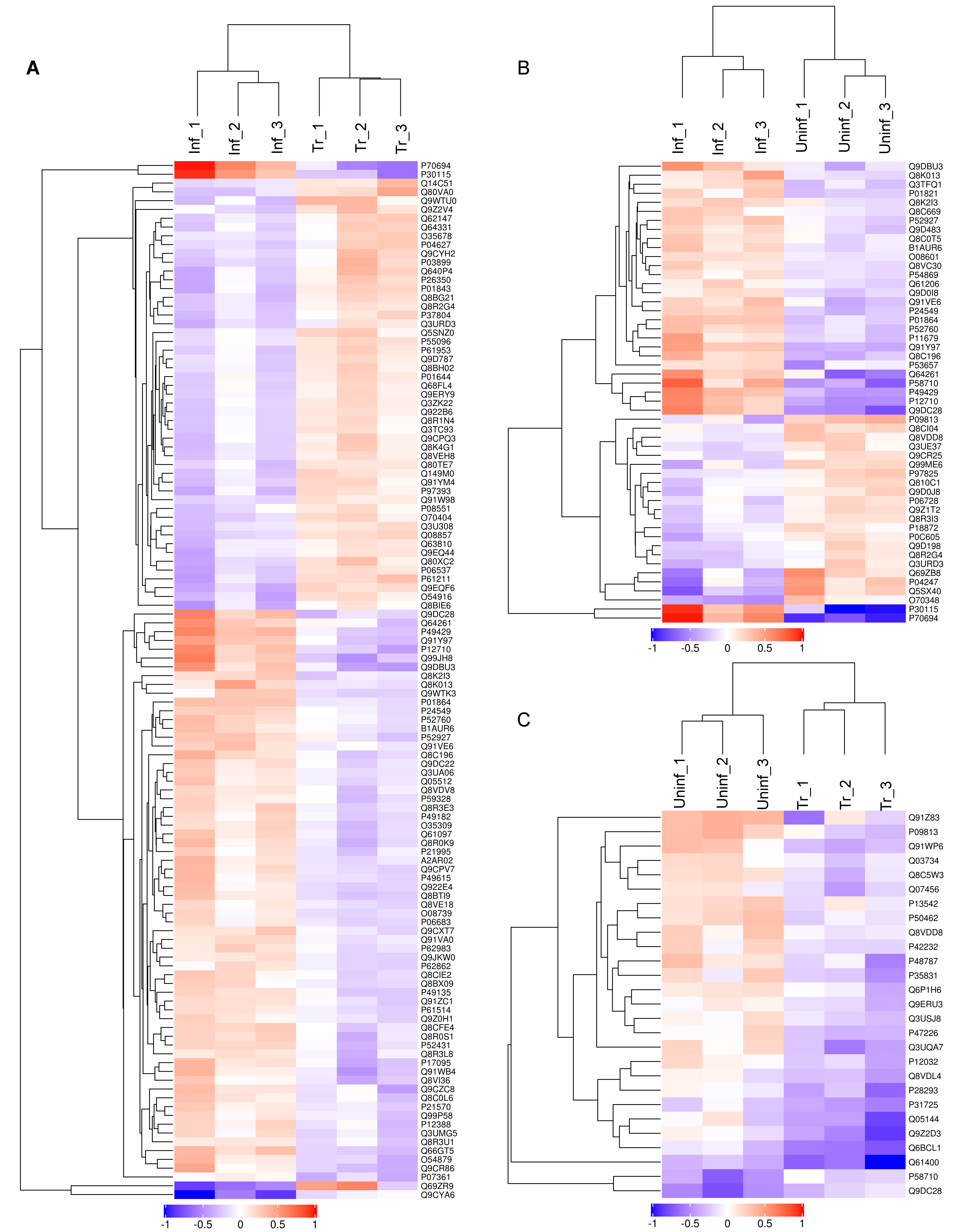

Supplement: Fig. S1 — Hierarchical clustering of differentially expressed proteins identified between (A) Inf vs Tr, (B) Inf vs Uninf, and (C) Uninf vs Tr. [file spectrum.04520-22-s0001.tif]

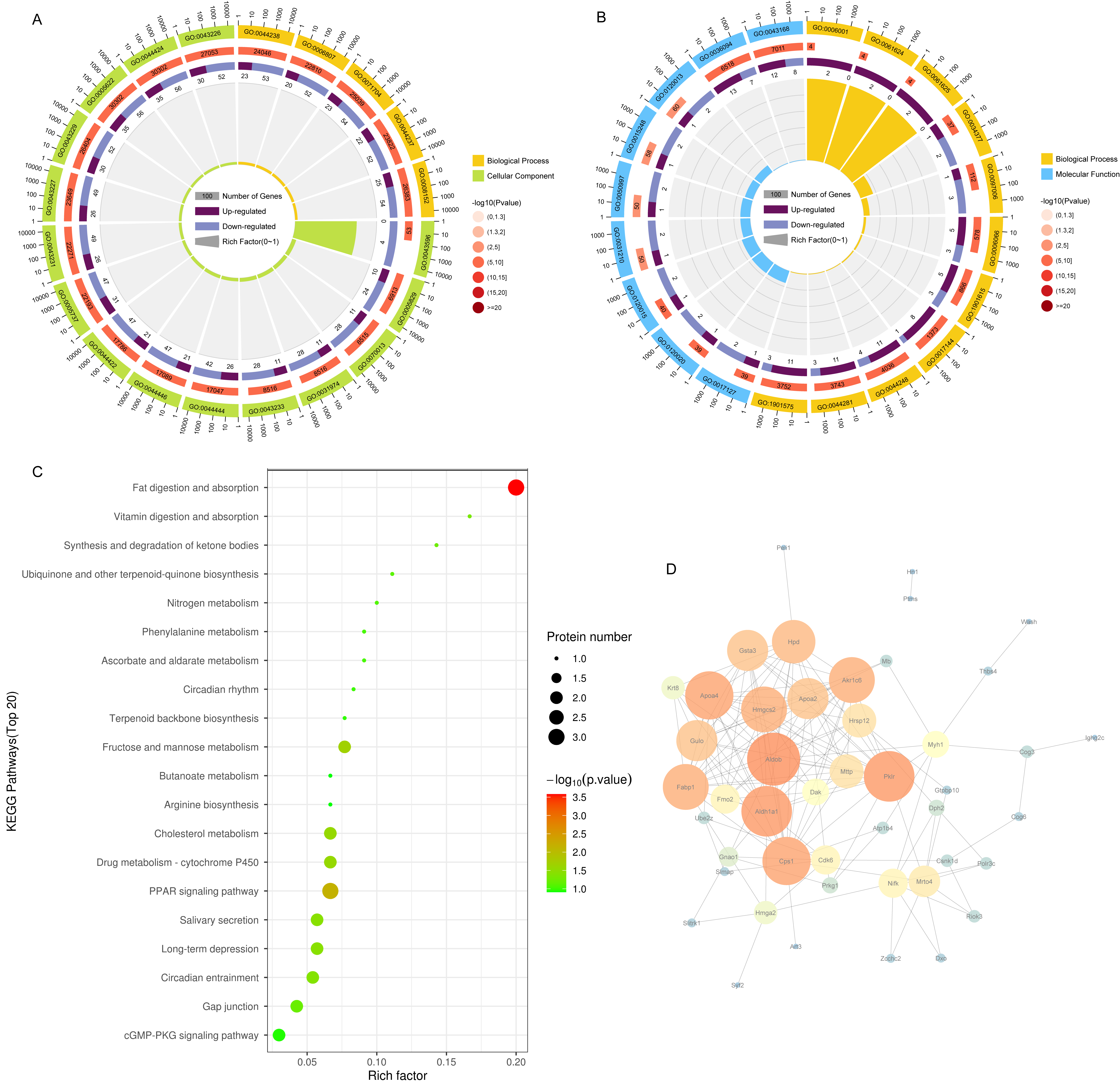

Supplement: Fig. S2 — Enrichment analysis of gene ontology and protein-protein interaction analysis. [file spectrum.04520-22-s0002.tif]

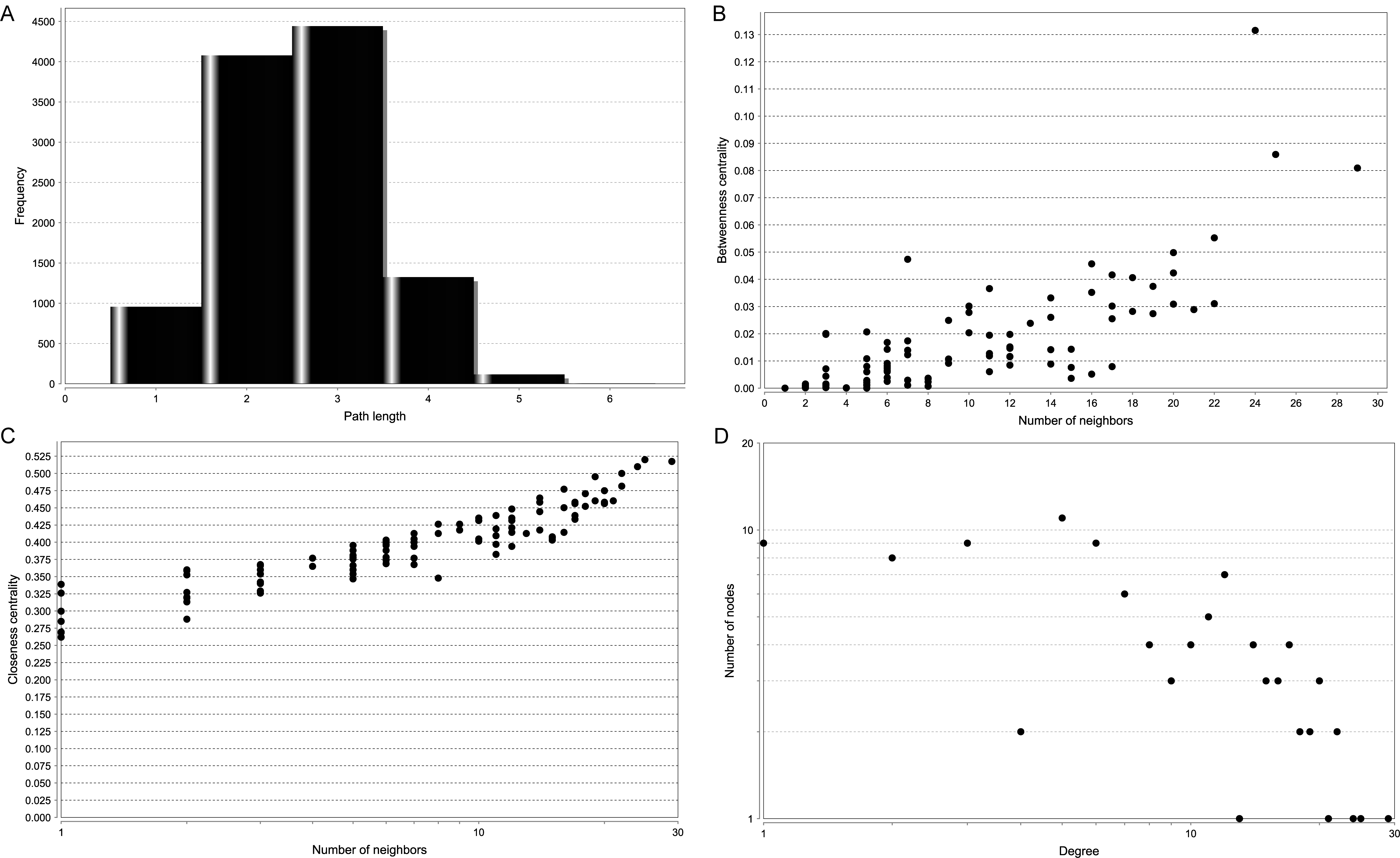

Supplement: Fig. S3 — Statistical analysis of the PPI network shown in Fig. 1D. [file spectrum.04520-22-s0003.tif]

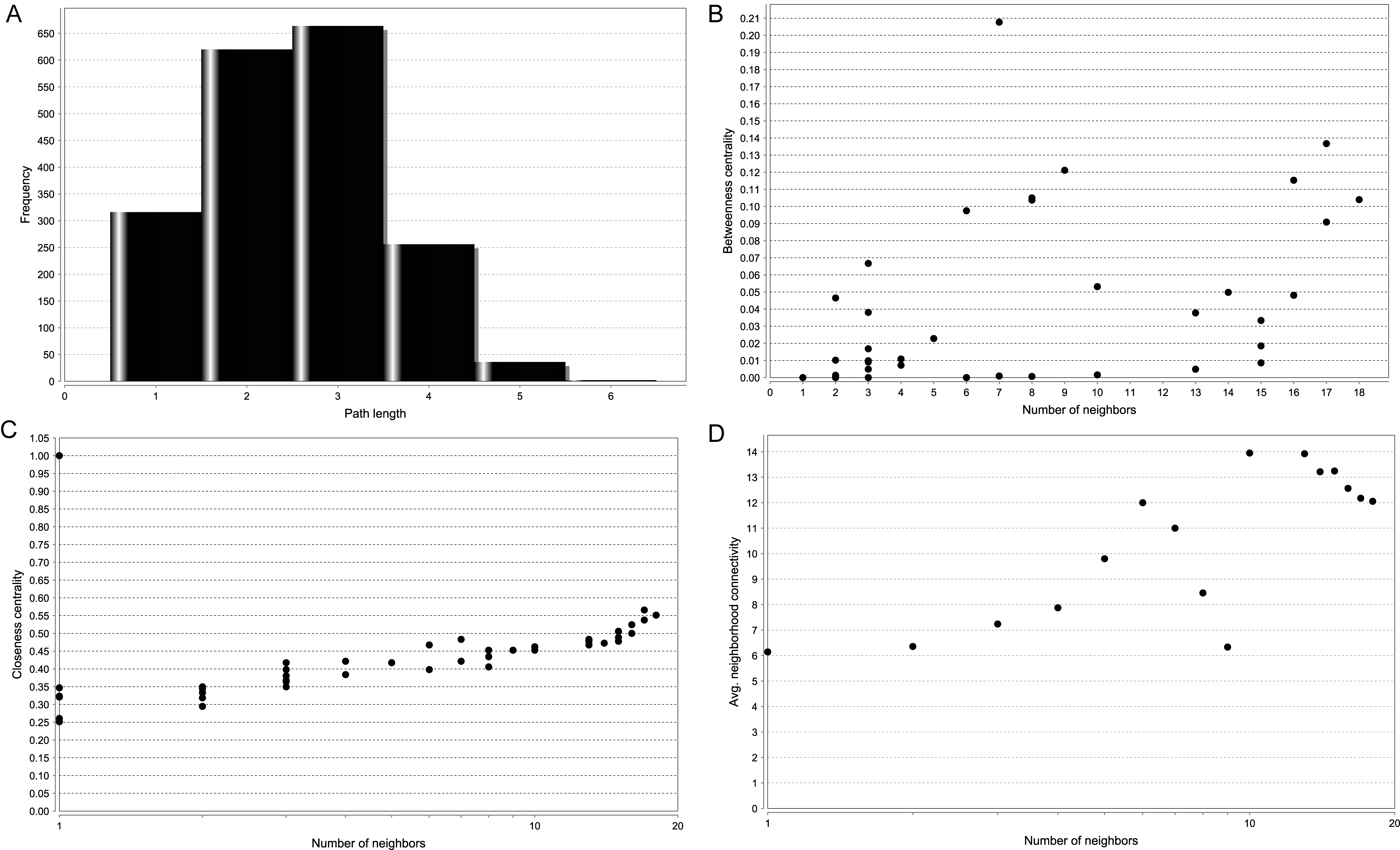

Supplement: Fig. S4 — Statistical analysis of the PPI network shown in Fig. S2D. [file spectrum.04520-22-s0004.tif]
