# Supplementary material for: Proteomic analysis of anti-MRSA activity of caerin 1.1/1.9 in a murine skin infection model and their in vitro anti-biofilm effects against Acinetobacter baumannii
Source: Microbiol Spectr. 2023 Oct 11;11(6):e04520-22. doi: 10.1128/spectrum.04520-22 (PMC10714828; doi:10.1128/spectrum.04520-22)
Supplement: Supplemental legends — Legends for Fig S1 to S4 and Tables S1 to S3. [file spectrum.04520-22-s0005.docx]

**Supplementary Figures and Tables**

**Figure S1** Hierarchical clustering of differentially expressed proteins identified between (A) Infected vs Treated, (B) Infected vs Uninfected, and (C) Uninfected vs Treated.

**Figure S2** Enrichment analysis of gene ontology and protein-protein interaction analysis. (A) Top 20 GO terms enriched by the DEPs upregulated in the Tr group with respect to the Inf group; (B) Top 20 GO terms enriched by the DEPs upregulated in the Inf group with respect to the Uninf group; (C) Top 20 KEGG pathways enriched by the DEPs of the Uninf group relative to the Inf group; (D) Protein-protein interactions between the DEPs of the Uninf group relative to the Inf group.

**Figure S3** Statistical analysis of the PPI network shown in **Figure 1D**.

**Figure S4** . Statistical analysis of the PPI network shown in **Figure S2D**

**Table S1** Comparison of protein profiles of different groups and supporting peptides identified by LC-MS/MS.

**Table S2** All supporting peptides identified by LC-MS/MS.

**Table S3** GSEA of the hallmark pathways enriched in the treatment with respect to the infected group.
